# Assessing Self-Reported Physical Activity Levels in Chronic Obstructive Pulmonary Disease (COPD) Patients: A Comprehensive Analysis Using the Behavioral Risk Factor Surveillance System-Web Enabled Analysis Tool (BRFSS-WEAT) Data

**DOI:** 10.7759/cureus.86206

**Published:** 2025-06-17

**Authors:** Jacques De Vos, Shruti Nair, Tim Mathew, Mehulkumar M Patel, Manaswini Chowdary Kaka

**Affiliations:** 1 Internal Medicine, Stellenbosch University, Cape Town, ZAF; 2 General Surgery, Lourdes Hospital, Kochi, IND; 3 Critical Care, Aster Medcity, Kochi, IND; 4 Internal Medicine, Ananya College of Medicine and Research, Kalol, IND; 5 Internal Medicine, American International Medical University, Saint Lucia, LCA

**Keywords:** brfss, chronic obstructive pulmonary disease (copd), demographic factors, healthcare access, physical activity, socioeconomic status

## Abstract

Background

Chronic obstructive pulmonary disease (COPD) includes chronic bronchitis and emphysema and is characterized by persistent airflow limitation. It is caused by long-term exposure to harmful particles or gases, leading to significant breathing difficulties and substantially impacting quality of life. Understanding the complexity and socioeconomic burden of COPD is vital for improving patient outcomes and addressing broader implications. This study assesses the self-reported physical activity levels of COPD patients in the United States using the 2021 Behavioral Risk Factor Surveillance System (BRFSS) data, exploring the impact of demographic, socioeconomic, and healthcare access variables.

Methodology

The 2021 BRFSS data were analysed, focusing on 34,061 individuals diagnosed with COPD, emphysema, or chronic bronchitis. Fisher’s exact test and the chi-square test were used to examine associations between self-reported physical activity levels and demographic (age, gender, race), socioeconomic (education, employment, income), and healthcare access (last routine check-up) variables, with 95% confidence intervals.

Results

Of the 435,780 BRFSS participants, 7.8% reported having COPD. Among these, 55% engaged in physical activity compared to 77.4% of non-COPD individuals. Physical activity levels were significantly lower among COPD patients (p < 0.001), with notable variations across age groups, genders, races, education levels, employment statuses, income categories, and time since the last routine check-up.

Conclusions

The findings reveal a significant disparity in physical activity between COPD patients and non-COPD individuals. These findings highlight the need for targeted interventions to improve physical activity among COPD patients to enhance their health outcomes and quality of life.

## Introduction

Chronic obstructive pulmonary disease (COPD) is a progressive respiratory illness that affects millions of people worldwide. With a high and increasing prevalence, COPD is a major global cause of death and morbidity. This condition is universally underdiagnosed and, as a result, often undertreated. This is especially true in the mild or early stages of the disease when doctors and patients are likely to underestimate its presence because symptoms do not yet interfere with daily activities [1]. Between 2011 and 2021, the overall prevalence of COPD in adults remained steady at 6.1% to 6.0%, as did the prevalence in most states and subgroups. However, the prevalence increased in adults aged over 75 years, those who lived in rural areas, and those who had ever smoked. The disparities between those who lived in rural areas and those who smoked grew [2].

The World Health Organization defines physical activity as any bodily movement produced by skeletal muscles that requires energy expenditure. Popular ways to be active include walking, cycling, wheeling, sports, active recreation, and play, which can be done at any level of skill and for enjoyment by everybody [3]. There is substantial evidence that regular physical activity can help lower the risk of numerous chronic diseases. On the other hand, a lack of physical activity poses a significant risk for poor outcomes in individuals with COPD and contributes to early mortality in patients with chronic illnesses [4-6].

The Behavioral Risk Factor Surveillance System (BRFSS) is a telephonic survey that is conducted each year. This study aims to assess the level of physical activity in patients with COPD using the BRFSS. We aim to assess the self-reported physical activity levels among COPD patients and examine their association with demographic, socioeconomic, and healthcare access variables.

## Materials and methods

Using the BRFSS database, a retrospective original research study was conducted. Data were extracted on June 25, 2024. As the BRFSS is a publicly accessible database containing de-identified information and does not directly involve human participants, ethics committee approval was not required [7].

Patients with a diagnosis of COPD, emphysema, or chronic bronchitis were included in this study (n = 34,061). The data were extracted from the BRFSS-Web-Enabled Analysis Tool (WEAT) for the year 2021, and 95% confidence intervals were reported. The prevalence of physical activity levels among these patients (exercises such as running, calisthenics, golf, gardening, or walking during the past month) in association with their demographic (age, gender, race), socioeconomic (education, employment, income), and healthcare care access (last routine check-up) status were included in the study. Essentially, any data that did not meet the inclusion criteria was excluded from the study.

The disease variable (CHCCOPD2) assessed whether participants had ever been told they had emphysema, chronic bronchitis, or COPD. Physical activity was measured using the variable EXERANY2, which asked if participants had engaged in any physical activity or exercise during the past month (e.g., walking, gardening, golfing, calisthenics, or running). These two variables (CHCCOPD2 and EXERANY2) were analyzed in combination to assess physical activity prevalence among individuals with COPD. Control variables, including age (calculated variable for four-level imputed age category, _AGE_G, 18-24, 25-44, 45-64, 65+), gender (SEX1), and race (calculated variable for four-level race, _RACEGR3), were examples of demographic characteristics. We analyzed the association between age, gender, and race with disease (CHCCOPD2) and exercise (EXERANY2). Socioeconomic characteristics were used as a control variable that included annual household income (INCOME3), employment status (EMPLOY1), and education level (EDUCA). Here, the income (INCOME3), employment status (EMPLOY1), and education level (EDUCA) were analyzed in relation to the disease (CHCCOPD2) and exercise variables (EXERANY2). Participants with missing values for primary variables (CHCCOPD2 or EXERANY2) were excluded from the analysis. Missing data for other covariates were not imputed. Healthcare access was assessed through the variable CHECKUP1, which indicated the time since the participant’s last routine check-up (within the past year, more than one year ago, or never). This was also analyzed in relation to the disease and exercise variables.

Descriptive statistics (frequencies and percentages) were generated using cross-tabulations in the BRFSS-WEAT tool. Data were stored in Microsoft Excel (Microsoft Corp., Redmond, WA, USA), and statistical analyses were performed using R version 4.3.1 (R Studio, Vienna, Austria). Fisher’s exact test and the chi-squared test were employed for categorical variables, depending on data distribution and sample size. A p-value <0.05 was considered statistically significant.

## Results

In 2021, in the United States, 435,780 people participated in the BRFSS study. Out of this, 34,061 (7.8%) self-identified or answered “Yes” to the question “Ever told you had chronic obstructive pulmonary disease, COPD, emphysema, or chronic bronchitis (CHCCOPD2).” These individuals were considered to have COPD.

In the past month, 18,739 (55%) participants with COPD were involved in physical activity, whereas 15,322 (45%) were not. Among participants who did not have COPD, 310,751 (77.4%) were involved and 90,968 (22.6%) were not involved in physical activity in the past month. The prevalence of COPD was significantly lower in participants engaged in physical activity in the past month, compared to those who were not (Table 1).

**Table 1:** Prevalence of self-reported COPD, emphysema, or chronic bronchitis by physical activity level. Values are mentioned as n (%). The chi-squared test was used. A p-value <0.05 was considered statistically significant. COPD: chronic obstructive pulmonary disease

COPD status (CHCCOPD2)	Physical activity (EXERANY2)	N	Percentage (%)	P-value	Chi-squared statistic
Yes	Active	18,739	55%	0.001*	8,495.524
Yes	Not active	15,322	45%	-	-
No	Active	310,751	77.40%	-	-
No	Not active	90,968	22.60%	-	-

The level of physical activity among patients with COPD was highest in the age group of 18-24 years (79%), male gender (58.5%), and Hispanic race (56.9%) and others (56.9%). The prevalence of COPD was significantly lower in participants involved in physical activity in the past month for all age groups, all genders, and all races, compared to those who were not (Table 2).

**Table 2:** Prevalence of self-reported COPD, emphysema, or chronic bronchitis by physical activity level based on demographic characteristics of the study participants. Values are mentioned as n (%). Fisher’s exact test was used. A p-value <0.05 was considered statistically significant. COPD: chronic obstructive pulmonary disease

Demographic group	COPD status (CHCCOPD2)	N	Physically active	Physically not active	P-value (Fisher’s exact test)
Age: 18–24 years	Yes	409	323 (79%)	86 (21%)	0.013*
Age: 18–24 years	No	25,452	21,305 (83.7%)	4,147 (16.3%)	-
Age: 25–44 years	Yes	3,028	2,065 (68.2%)	963 (31.8%)	<0.001*
Age: 25–44 years	No	102,653	84,031 (81.9%)	18,622 (18.1%)	-
Age: 45–64 years	Yes	11,974	6,401 (53.5%)	5,573 (46.5%)	<0.001*
Age: 45–64 years	No	137,933	107,004 (77.6%)	30,929 (22.4%)	-
Age: 65+ years	Yes	18,650	9,950 (53.4%)	8,700 (46.6%)	<0.001*
Age: 65+ years	No	135,681	98,411 (72.5%)	37,270 (27.5%)	-
Gender: Male	Yes	14,125	8,261 (58.5%)	5,864 (41.5%)	<0.001*
Gender: Male	No	188,116	150,113 (79.8%)	38,003 (20.2%)	-
Gender: Female	Yes	19,936	10,478 (52.6%)	9,458 (47.4%)	<0.001*
Gender: Female	No	213,603	160,638 (75.2%)	52,965 (24.8%)	-
Race: White, non-Hispanic	Yes	27,014	14,798 (54.8%)	12,216 (45.2%)	<0.001*
Race: White, non-Hispanic	No	293,898	232,119 (79%)	61,779 (21%)	-
Race: Black, non-Hispanic	Yes	2,352	1,251 (53.2%)	1,101 (46.8%)	<0.001*
Race: Black, non-Hispanic	No	30,181	21,462 (71.1%)	8,719 (28.9%)	-
Race: Hispanic	Yes	1,564	890 (56.9%)	674 (43.1%)	<0.001*
Race: Hispanic	No	36,613	25,329 (69.2%)	11,284 (30.8%)	-
Race: Other	Yes	2,273	1,293 (56.9%)	980 (43.1%)	<0.001*
Race: Other	No	31,264	24,316 (77.8%)	6,948 (22.2%)	-

The level of physical activity among patients with COPD was the highest in participants with advanced education level (61.1%), employed (66.6%), and high income (76.7%) categories. The prevalence of COPD was significantly lower in participants involved in physical activity in the past month for all education levels, all employment statuses, and all income categories, compared to those who were not (Table 3).

**Table 3:** Prevalence of self-reported COPD, emphysema, or chronic bronchitis by physical activity level based on socioeconomic characteristics of the study participants. Values are mentioned as n (%). Fisher’s exact test was used. A p-value <0.05 was considered statistically significant. COPD: chronic obstructive pulmonary disease

Socioeconomic characteristics	COPD status (CHCCOPD2)	N	Physically active	Physically not active	P-value (Fisher’s exact test)
Basic education	Yes	15,392	7,335 (47.7%)	8,057 (52.3%)	<0.001*
Basic education	No	120,925	79,758 (66%)	41,167 (34%)	-
Advanced education	Yes	18,540	11,337 (61.1%)	7,203 (38.9%)	<0.001*
Advanced education	No	278,644	229,430 (82.3%)	49,214 (17.7%)	-
Employed	Yes	7,851	5,228 (66.6%)	2,623 (33.4%)	<0.001*
Employed	No	215,059	175,680 (81.7%)	39,379 (18.3%)	-
Not employed	Yes	25,699	13,243 (51.5%)	12,456 (48.5%)	<0.001*
Not employed	No	179,204	129,479 (72.3%)	49,725 (27.7%)	-
Low-income	Yes	18,843	9,525 (50.5%)	9,318 (49.5%)	<0.001*
Low-income	No	130,740	89,467 (68.4%)	41,273 (31.6%)	-
Mid-income $50,000 to $150,000/year	Yes	7,297	4,840 (66.3%)	2,457 (33.7%)	<0.001*
Mid-income $50,000 to $150,001/year	No	147,117	122,689 (83.4%)	24,428 (16.6%)	-
High-income >$150,000/year	Yes	806	618 (76.7%)	188 (23.3%)	<0.001*
High-income >$150,001/year	No	37,847	34,516 (91.2%)	3,331 (8.8%)	-

Among participants with routine check-ups within the past year, 15,854 (54.2%) participants with COPD were physically active compared to 235,034 (76.8%) participants without COPD. Among participants with routine check-ups more than one year, 2,677 (61.1%) participants with COPD were physically active compared to 72,124 (79.6%) participants without COPD. Irrespective of time since last routine check-up (within the last year or more than a year ago), physical activity in the last month was significantly associated with lower prevalence of COPD among the participants (Table 4).

**Table 4:** Prevalence of self-reported COPD, emphysema, or chronic bronchitis by physical activity level based on time since last routine check-up. Values are mentioned as n (%). Fisher’s exact test was used. A p-value <0.05 was considered statistically significant. COPD: chronic obstructive pulmonary disease

Time since last routine check-up	COPD status (CHCCOPD2)	N	Physically active	Physically not active	P value (Fisher’s exact test)
Within the past year	Yes	29,241	15,854 (54.2%)	13,387 (45.8%)	<0.001*
Within the past year	No	305,953	235,034 (76.8%)	70,919 (23.2%)	-
More than a year ago or never	Yes	4,380	2,677 (61.1%)	1,703 (38.9%)	<0.001*
More than a year ago or never	No	90,558	72,124 (79.6%)	18,434 (20.4%)	-

## Discussion

A retrospective study was conducted in June 2024 to assess activity levels among patients with COPD in the United States, using the BRFSS database.

The effects of COPD include cardiovascular comorbidities, muscle wasting, and osteoporosis that ultimately affect the patient’s level of activity. This can impact quality of life as an inability to exercise exacerbates the patient’s overall health condition [8]. Pulmonary rehabilitation using physical exercise has the potential to enhance the patient’s exercise abilities, quality of life, and symptomatology, even in severe COPD [9]. For those living with COPD and frailty, multidisciplinary, individualized, and group-based approaches, together with flexible service delivery, have been shown to improve the value of exercise-based treatments, though efforts are generally required to encourage activity completion [10,11]. Exercise limitation and physical inactivity, although being separate yet related concepts, both frequently present in those suffering from COPD and contribute to the disease burden. They can independently predict negative outcomes in such patients and are therefore beneficial when assessing a patient with COPD. The six-minute walk test and the incremental and endurance shuttle walk tests, which are not resource-demanding, can be used to test a patient’s physical capacity. Their results correlate with negative COPD outcomes [12]. Aerobic exercise demonstrates benefits that include decreased hyperinflation, decreased exertional dyspnea, enhanced exercise tolerance, better quality of life, fewer COPD exacerbations, and fewer sick days taken [13]. Besides aerobic training, various exercise modalities have been utilized in those with COPD, including resistance training, balance training, whole body vibration training, and more. In the end, the type of exercise chosen is dependent on individual limitations, comorbidities, preferences, needs, and personal goals. Resources, equipment, trainer expertise, and costs of programs also come into play [14].

In this study, participants who answered yes to the question of whether they were told that they have COPD, emphysema, or chronic bronchitis will be considered to have COPD. As shown in Table 1, during the last month, 18,739 (55%) participants with COPD were involved in physical activities, whereas 310,751 (77.4%) participants without COPD were involved in physical activities. The chi-squared test resulted in a p-value of less than 0.05, showing a significant association between physical activity during the last month and the prevalence of COPD. The prevalence of COPD was lower in those participating in physical activities during the last month compared to those who did not engage in such activities. Lower physical activity has been predictive of COPD exacerbations in Horner et al. [15]. Even with regular treatment, most COPD patients with moderate-to-severe disease have a lifestyle of low physical activity [16].

As shown in Table 2, the prevalence of physical activity during the last month was highest in the age group of 18-24 years, male gender, and white race. With increasing age, physical activity during the last month in those with COPD decreased. From 323 (79%) in participants in the age group of 18-24 years, to 2,065 (68.2%) in the age group of 25-44 years, to 6,401 (53.5%) in the age group of 45-64 years, to 9,950 (53.4%) in those aged over 65 years. The prevalence of physical activity in the last month in those with COPD was nearly similar across genders, 8,261 (58.5%) in males and 10,478 (52.6%) in females. It was also similar across all races. There was a statistically significant association between physical activity in the last month and lower prevalence of COPD across all age groups, races, and genders. Wan et al. [17] demonstrated the predictability of exercise capacity and physical activity in patients with COPD using epigenetic measures of biological age.

As shown in Table 3, the prevalence of physical activity during the last month was the highest in participants with an advanced education, the employed, and high-income earners. The prevalence of physical activity during the last month in those with COPD was higher in participants with an advanced education, 11,337 (61.1%), compared to 7,335 (47.7%) participants with a basic education. The prevalence of physical activity during the last month in those with COPD was also higher in employed participants, 5,228 (66.6%), compared to those unemployed, 13,243 (51.5%). The prevalence of physical activity during the last month in those with COPD increased with higher incomes across participants. From 9,525 (50.5%) of low-income earners, to 4,840 (66.3%) of mid-income earners, to 618 (76.7%) of high-income earners. There was a statistically significant association between physical activity in the last month and lower prevalence of COPD across education level, employment status, and annual income.

As shown in Table 4, the prevalence of physical activity during the last month in participants with COPD was higher in those who never had a routine check-up, or more than a year ago, 2,677 (61.1%), compared to those who had a routine check-up within the past one year, 15,854 (54.2%). There was a statistically significant association between physical activity in the last month and lower prevalence of COPD among those who had a routine check-up within the past year, as well as among those who never had a routine check-up (or more than a year ago).

Limitations

The BRFSS dataset does not indicate the type or severity of COPD, nor how many months prior the participants developed the disease, nor any consequences thereof. These may affect the physical activity of participants. Interview questions were asked related only to physical activity level in the last month. It is uncertain if one month of physical activity would cause a significant improvement in COPD prognosis. Establishing the exact onset of disease and the timeline as it pertains to physical activity levels is also difficult.

Diagnosis was based on self-reported data, rather than on history taken and examinations performed in a clinical setting. Telephonic surveys were conducted, rather than in-person surveys. Thus, those without access to phones, not using phones, or those simply unavailable were not contacted. As surveys were conducted in English and a limited number of other languages, those unable to speak these languages may belong to underrepresented groups and were excluded.

This study was a retrospective observational study, and thus, only association, as opposed to causation, can be established. Prospective research studies are needed to determine if physical activity can assist in preventing or reducing the risk of COPD.

## Conclusions

This study highlights a significant disparity in physical activity levels between individuals with and without COPD in the United States, as evidenced by nationally representative BRFSS data. Lower levels of self-reported physical activity were consistently associated with COPD across all demographic, socioeconomic, and healthcare access variables. While the study did not establish causality due to its cross-sectional design, the strong associations suggest that physical inactivity is a critical concern among COPD patients. The study did not adjust for other comorbidities such as cardiovascular, musculoskeletal, or neurological conditions that may independently influence physical activity levels. These findings underscore the need for targeted, equitable public health interventions aimed at improving physical activity in this population. Future prospective studies are warranted to better understand the causal relationship and guide evidence-based recommendations for physical activity in COPD management.
